# Supplementary material for: Accuracy of frozen section in intraoperative margin assessment for breast-conserving surgery: A systematic review and meta-analysis
Source: PLoS One. 2021 Mar 18;16(3):e0248768. doi: 10.1371/journal.pone.0248768 (PMC7971883; doi:10.1371/journal.pone.0248768)
Supplement: Attachment — Submitted filename: Response to Reviewers.docx [file pone.0248768.s004.docx]

1. Please ensure that your manuscript meets PLOS ONE's style requirements, including those for file naming. The PLOS ONE style templates can be found at

<https://journals.plos.org/plosone/s/file?id=wjVg/PLOSOne_formatting_sample_main_body.pdf>

and

<https://journals.plos.org/plosone/s/file?id=ba62/PLOSOne_formatting_sample_title_authors_affiliations.pdf>

**Thank you very much for your consideration.**

2. We suggest you thoroughly copyedit your manuscript for language usage, spelling, and grammar. If you do not know anyone who can help you do this, you may wish to consider employing a professional scientific editing service.

Whilst you may use any professional scientific editing service of your choice, PLOS has partnered with both American Journal Experts (AJE) and Editage to provide discounted services to PLOS authors. Both organizations have experience helping authors meet PLOS guidelines and can provide language editing, translation, manuscript formatting, and figure formatting to ensure your manuscript meets our submission guidelines. To take advantage of our partnership with AJE, visit the AJE website (http://learn.aje.com/plos/) for a 15% discount off AJE services. To take advantage of our partnership with Editage, visit the Editage website ([www.editage.com](http://www.editage.com/)) and enter referral code PLOSEDIT for a 15% discount off Editage services.  If the PLOS editorial team finds any language issues in text that either AJE or Editage has edited, the service provider will re-edit the text for free.

Upon resubmission, please provide the following:

- The name of the colleague or the details of the professional service that edited your manuscript

**Maria Carolina Rodrigues**

- A copy of your manuscript showing your changes by either highlighting them or using track changes (uploaded as a *supporting information* file)
- A clean copy of the edited manuscript (uploaded as the new *manuscript* file)

3. Please confirm that you have included all items recommended in the PRISMA checklist including the full electronic search strategy used to identify studies with all search terms and limits for at least one database.

**We performed a new review on the Prisma checklist.**

4. Please described the data extraction methods in more details. We would expect to see reporting of the specific information extracted from the manuscripts.

**We improved the item 2.3 Data extraction:**

**Two researchers manually extracted the following data from all studies included in this review:  number of patients, number of cases, staging, age, concept of free margin, intraoperative margin assessment method, follow-up time, number of true positives (frozen section and paraffin with positive margins), number of true negatives (frozen section and paraffin with free margins), number of false positives (positive frozen section margins and free paraffin margins), number of false negatives (free frozen section margins and positive paraffin margins), total positive cases with the paraffin method, total negative cases with the paraffin method and re-excision rate. For local recurrence and overall survival, data was combined using the inverse variance method on the log-HR scale, and on the log-RR scale for dichotomous outcomes. If the data were diverse enough to permit effect sizes combination in a meaningful or valid manner, we presented such results individually using table and graphical formats, as well as a narrative approach to summarize the data. In cases where accuracy was not explicitly reflected, we constructed a 2 x 2 table to calculate the required data. All disagreements were resolved via consensus by a third senior reviewer.**

**We provide our data extraction table. You can find it in the support information file. (S1_table1).**

5. Please include your tables as part of your main manuscript and remove the individual files. Please note that supplementary tables should be uploaded as separate "supporting information" files.

**Thank you very much for your consideration.**

6. Please include captions for your Supporting Information files at the end of your manuscript, and update any in-text citations to match accordingly. Please see our Supporting Information guidelines for more information: <http://journals.plos.org/plosone/s/supporting-information>.

**Thank you very much for your consideration.**

Additional Editor Comments:

This is a well-designed systematic review and meta-analysis. The findings are not very novel as one of the reviewers commented, while its method is overall acceptable. See the following concerns for your consideration.

Major points:

1. The search terms "lumpectomy" and "breast conserving surgery" are missing but should be included.

**The search terms "lumpectomy" and "breast conserving surgery" are included at MESH tree in PubMed for segmental mastectomy**.

2. Does frozen section also have high sensitivity? If so, please comment on it and add "sensitivity" in the concluding sentence.

**After the sensitivity analysis considering only the cohort studies, the test's sensitivity increased from 80 to 90%.  We ended up at the conclusion:**

**Frozen section is a reliable technique for intraoperative margin assessment in breast-conserving surgery with high levels of accuracy, sensitivity and specificity. Due to this high precision for negative results, routine use of this test may aid surgeons in the pursuit of tumor-free surgical margins, therefore reducing reoperation rates.**

3. Cross-sectional and cohort studies have different study design and scientific rigor/biases. It will be very important and interesting to conduct subgroup analyses based on study design (Cross-sectional and cohort studies in different groups).

**We performed analyses considering only cohort and cross-sectional studies. There was an increase in sensitivity. We described it in the text and added the figures (3.1.1 Sensitivity and subgroup analysis):**

**A sensitivity analysis was carried out considering only the cohort studies. Sensitivity and specificity were evaluated in 11 studies(15,16,22–29,31). Intraoperative assessment sensitivity was 0.87, with a CI of 0.85–0.89, p=0.0000, and inconsistency (I^2^) of 86.6%, which included a total of 4228 tests (Figure 5). Specificity was 0.97, with a CI of 0.96–0.97, p=0.0000, and I^2^ of 90.8% in the same sample (Figure 6). The accuracy, represented by the area under the SROC curve, is 0.98 (Figure 7).**

**A sensitivity analysiss was also carried out considering only the cross-section studies. Sensitivity and specificity were evaluated in 6 studies(32–37). Intraoperative assessment sensitivity was 0.64, with a CI of 0.59–0.69), p=0.0000, and inconsistency (I^2^) of 97.1%, which included a total of 1387tests (Figure 8). Specificity was 0.98, with a CI of 0.97–0.99, p=0.0000, and I^2^ of 91.5%, in the same sample (Figure 9). The accuracy, represented by the area under the SROC curve, is 0,98 (Figure 10).**

4. Histology types of breast cancer may influence its outcome, and cancer distribution pattern. So it may also be necessary either discuss this point in light of/citing prior studies ((e.g. PMID: 28746732 and PMID: 30518616) or conduct subgroup analysis.

**Unfortunately, the studies did not allow the extraction of individual data about accuracy by histological type. Only 2 authors performed an evaluation by histological type. We described it in the results. The authors Yang 2017 and Metzger-Filho 2019 described the overall survival versus histological data.**

**We added a paragraph at 3.1.1 Sensitivity and subgroup analysis:**

**The sensibility analysis by histological subtype was not possible due to lack of individual data on each test. Only two authors performed an evaluation by histological type, which will be describe in the results. Osako *et al*. showed an increase of 11.9 chance of positive margins in the final pathology (p=0.01) in patients with invasive lobular carcinoma, larger tumors, or extensive intraductal component (EIC), and who were 50 years old or younger. Jorn *et al*. claimed that only disease multifocality (histologically discrete tumors at least 2 cm apart) could be a risk factor to increased reoperation rates, with OR of 3.41 (CI 1.38-8.40, p=0.008). The article did not associate histological subtype and tumor sizer with further surgeries. The invasive ductal carcinoma subtype had an OR of 0.75 (CI 0.31-1.82, p=0.37), invasive lobular carcinoma subtype had an OR of 2.29 (CI 0.52-9.98, p=0.37) and larger tumor size (> 2 cm) OR 1.33 (CI 0.26-6.74, p=0.733).**

Minor points:

1. It will be helpful if the sensitivity and specificity of each study are shown.

**These were shown at figure 2 e 3.**

2. Recommend to cite the papers that shows increasing use of lumpectomy/BCS in recent years (e.g. PMID: 24929768, PMID: 29423512 and PMID: 28586788)

**The PMID: 24929768 was added in introduction:**

**Data from a cohort study, which included 24,217 patients, showed those that did not use frozen section during surgical procedures were four times more likely to need reoperation than women who underwent a lumpectomy for breast cancer followed by a frozen section procedure. Despite the advantages of macroscopic analysis, this procedure can be performed directly by the surgeon, and boasts of higher accuracy (80%), sensitivity (49%) and specificity (86%) than other techniques**

**The PMID: 28586788 and PMID: 29423512 was added in discussion:**

**Since 2013, a trend in the reduction of reoperation rates has been observed, which was described by Yang *et al*. Therefore, in 2016, Morrow *et al*. showed a decrease of 16% in re-excision rates among surgeons consensus.**

3. Writing style issues: Just some examples.

Title: in breast ... may be replaced with for breast...

**Ok. Thank you.**

Abstract: metanalysis and exams may be replaced with meta-analysis and studies, respectively; CI should be spelled out, I2 may be replaced with I-squared or I-2;

**Ok. Thank you**.

**We preferred replaced exam to test, to prevent studies = papers due to our unit analysis was performed by test and not only by number the included patient.**

Section 2.2: "we admitted" may be replaced with "we included"

**Ok. Thank you.**

Section 2.4: a "that" is needed before "were selected"

**Ok. Thank you.**

I would also modify the abstract as:

Background and objectives: It is well established that tumor-free margins is an important factor for reducing local recurrence and reoperation rates. This systematic review with meta-analysis of frozen section intraoperative margin assessment aims to evaluate the accuracy, and reoperation and survival rates, and to establish its importance in breast-conserving surgery. Methods: A thorough review was conducted in all online publication-databases for the related literature up to March 2020. MeSH terms used: “Breast Cancer”, “Segmental Mastectomy” and “Frozen Section”. We included the studies that evaluated accuracy of frozen section, reoperation and survival rates. To ensure quality of the included articles, the QUADAS-2 tool (adapted) was employed. The assessment of publication bias by graphical and statistical methods was performed using the funnel plot and the Egger’s test. The review protocol was registered in PROSPERO (CRD42019125682). Results: Nineteen studies were deemed suitable, with a total of 6,769 cases. The reoperation rate on average was 5.9%. Sensitivity was 0.81, with a Confidence Interval of 0.79–0.83, p=0.0000, I2=95.1%, and specificity was 0.97, with a Confidence Interval of 0.97–0.98, p=0.0000, I-2 =90.8%, for 17 studies and 5,615 cases. Accuracy was 0.98. Twelve studies described local recurrence and the highest cumulative recurrence rate in 3 years was 7.5%. The quality of the included studies based on the QUADAS-2 tool showed a low risk of bias. There is no publication bias (p=0.32) and the funnel plot showed symmetry. Conclusion: Frozen section is a reliable procedure with high accuracy, sensitivity and specificity in intraoperative margin assessment of breast-conserving surgery. Therefore, this modality of margin assessment could be useful in reducing reoperation rates.

Keywords: breast cancer; conserving surgery; frozen section; diagnostic accuracy; margin assessment; intraoperative test

[Note: HTML markup is below. Please do not edit.]

Reviewers' comments:

Reviewer's Responses to Questions

**Comments to the Author**

1. Is the manuscript technically sound, and do the data support the conclusions?

The manuscript must describe a technically sound piece of scientific research with data that supports the conclusions. Experiments must have been conducted rigorously, with appropriate controls, replication, and sample sizes. The conclusions must be drawn appropriately based on the data presented.

Reviewer #1: Partly

Reviewer #2: Partly

2. Has the statistical analysis been performed appropriately and rigorously?

Reviewer #1: I Don't Know

Reviewer #2: N/A

3. Have the authors made all data underlying the findings in their manuscript fully available?

The [PLOS Data policy](http://www.plosone.org/static/policies.action#sharing) requires authors to make all data underlying the findings described in their manuscript fully available without restriction, with rare exception (please refer to the Data Availability Statement in the manuscript PDF file). The data should be provided as part of the manuscript or its supporting information, or deposited to a public repository. For example, in addition to summary statistics, the data points behind means, medians and variance measures should be available. If there are restrictions on publicly sharing data—e.g. participant privacy or use of data from a third party—those must be specified.

Reviewer #1: Yes

Reviewer #2: Yes

4. Is the manuscript presented in an intelligible fashion and written in standard English?

PLOS ONE does not copyedit accepted manuscripts, so the language in submitted articles must be clear, correct, and unambiguous. Any typographical or grammatical errors should be corrected at revision, so please note any specific errors here.

Reviewer #1: No

Reviewer #2: Yes

5. Review Comments to the Author

Please use the space provided to explain your answers to the questions above. You may also include additional comments for the author, including concerns about dual publication, research ethics, or publication ethics. (Please upload your review as an attachment if it exceeds 20,000 characters)

Reviewer #1: This manuscript demonstrated results of systematic review and metanalysis on accuracy of frozen section on margin evaluation in breast conserving surgery.

1. This topic is not cutting edge; it was extensively discussed on the literature more than 10 years ago on this breast pathology practice. Currently, the frozen section for margin evaluation in partial mastectomy specimens is not commonly used in an intraoperative setting of pathology practice at least in hospitals located in the US due to some technical issues including but not limited to the following:
-It is well known that breast tissue is fatty and difficult to cut into a complete section for microscopic evaluation when it is fresh (without fixed in formalin solution for certain period of time). This nature of fresh breast tissue makes margin evaluation by frozen section often challenging. Nowadays, some surgeons use radiographic image on fresh tissue specimen instead of frozen sections to decide if margins are clear and if a new margin (or which margin) needs
to be re-excised before completing a breast conserving surgery. This practice has been gone well over the years in our institution.

-Also sometimes cutting frozen section on fresh breast tissue takes a longer time due to fatty nature of the specimen compared to cutting non-fatty tissues; furthermore the frozen section practice prolongs operating time. Although this study showed an accuracy of frozen section in intra-operative margin assessment in breast conserving surgery, now this practice has not been widely adopted at least in the US, probably, partly due to the reasons mentioned above. Therefore, not sure if this study had significant clinical utility at the current breast conserving surgery practice across the world.

**Thank you for your consideration. We know that frozen section is not a common procedure for breast cancer in United States, but it is still use worldwide. Data from a cohort study, including 24.217 patients, showed four times more chance of reoperation without frozen section during surgical procedures (American College of Surgeons National Surgical Quality Improvement Program – NSQIP) than women who undergo a lumpectomy for breast cancer surgery with frozen section exam (Mayo Clinic Rochester). We added it in Introduction.**

2. A total of 19 studies (42 references) were analyzed; not sure if number of the studies is enough for this metanalysis.

**We perform a rigorous search strategy to find the studies related to the frozen section in breast cancer surgery. We are confident that this search strategy covers all studies that could have available data to perform it. Here we have 19 studies including 6760 participants.**

3. English needs to be polished; there are some grammatical errors.

**Thank you. We performed a grammar review with a specialist.**

Reviewer #2:

1. Some hospitals routinely perform intraoperative margin assessment in breast conserving surgery, while others do not. Can you compare advantage and disadvantage of different techniques, including gross analysis, radiology, and cytology examination? How is local recurrence and reoperation rate individually? What scenarios do authors prefer to do frozen sections rather than other methods?

**We added the paragraph in Introduction:**

**Despite the advantages of macroscopic analysis, this procedure can be performed directly by the surgeon, and boasts of higher accuracy (80%), sensitivity (49%) and specificity (86%) than other techniques.**

2. Is there any difference about false positive rate and false negative rate among different cancer type, such as IDC, ILC or mucinous carcinoma?

**We added a paragraph at 3.1.1 Sensitivity and subgroup analysis:**

**The sensibility analysis by histological subtype was not possible due to lack of individual data on each test. Only two authors performed an evaluation by histological type, which will be describe in the results. Osako *et al*. showed an increase of 11.9 chance of positive margins in the final pathology (p=0.01) in patients with invasive lobular carcinoma, larger tumors, or extensive intraductal component (EIC), and who were 50 years old or younger. Jorn *et al*. claimed that only disease multifocality (histologically discrete tumors at least 2 cm apart) could be a risk factor to increased reoperation rates, with OR of 3.41 (CI 1.38-8.40, p=0.008). The article did not associate histological subtype and tumor sizer with further surgeries. The invasive ductal carcinoma subtype had an OR of 0.75 (CI 0.31-1.82, p=0.37), invasive lobular carcinoma subtype had an OR of 2.29 (CI 0.52-9.98, p=0.37) and larger tumor size (> 2 cm) OR 1.33 (CI 0.26-6.74, p=0.733).**

3. There is the large variability in false negative rate from 0% to 23%. What are reasons to cause this?

**The reason for the large variability could be associated with the pathologist's experience. It is not possible to identify in the studies the physician's expertise.
Moreover, a different definition of free margin between studies could explain this large variability.**

4. In Jorns and Ko studies, the number of patients with reoperation is larger than the number of patients with false negative rates. Except false negative margin, is there any other reason to let surgeons re-operate patients?

**Jorns study: 11 patients were reoperated. Among those 5 were true positive and 6 were false negative at frozen section which led the patient to another surgery. Based on this all patients were submitted to another surgery based only in the frozen section exam.**

**Ko study: considered some frozen sections as undetermined margins. This kind of margins were not evaluated even as positive neither negative. This factor increases the number of reoperation rate.**

5. Is it possible to list turnaround time for results in the table considering that one of disadvantages of intraoperative assessment is to increase surgery time?

**Eleven out of 19 studies described turnaround time to perform the frozen section exam, it ranges from 10 to 50 minutes.**

**We added a column in table 1.**

| **Author** | **Turnaround time** |
| --- | --- |
| **Anila 2016** | 20 min |
| **Caruso 2011** | 20 min |
| **Cendán 2005** | 13 min |
| **Dener 2009** | 25 min |
| **Ikeda 1997** | - |
| **Jorns 2014** | 24 min |
| **Kikuyama 2015** | - |
| **Kim 2016** | - |
| **Ko 2017** | 40 min |
| **Noguchi 1995** | - |
| **Nowikiewicz 2019** | 15 min |
| **Olson 2007** | 25 min |
| **Osako 2015** | 50 min |
| **Pinotti 2002** | - |
| **Riedl 2008** | 20-30 min |
| **Rusby 2008** | 10 -20 min |
| **Sauter 1994** | - |
| **Tan 2014** | - |
| **Weber 2008** | - |

6. PLOS authors have the option to publish the peer review history of their article ([what does this mean?](https://journals.plos.org/plosone/s/editorial-and-peer-review-process#loc-peer-review-history)). If published, this will include your full peer review and any attached files.


If you choose “no”, your identity will remain anonymous but your review may still be made public.


**Do you want your identity to be public for this peer review?** For information about this choice, including consent withdrawal, please see our [Privacy Policy](https://www.plos.org/privacy-policy).

Reviewer #1: No

Reviewer #2: No

[NOTE: If reviewer comments were submitted as an attachment file, they will be attached to this email and accessible via the submission site. Please log into your account, locate the manuscript record, and check for the action link "View Attachments". If this link does not appear, there are no attachment files.]

While revising your submission, please upload your figure files to the Preflight Analysis and Conversion Engine (PACE) digital diagnostic tool, <https://pacev2.apexcovantage.com/>. PACE helps ensure that figures meet PLOS requirements. To use PACE, you must first register as a user. Registration is free. Then, login and navigate to the UPLOAD tab, where you will find detailed instructions on how to use the tool. If you encounter any issues or have any questions when using PACE, please email PLOS at [figures@plos.org](mailto:figures@plos.org). Please note that Supporting Information files do not need this step.

*In compliance with data protection regulations, you may request that we remove your personal registration details at any time.* [*(Remove my information/details)*](https://www.editorialmanager.com/pone/login.asp?a=r)*. Please contact the publication office if you have any questions.*
